# Study of the Relationship between Entropy and Hardness in Laser Cutting of Hardox Steel

**DOI:** 10.3390/ma16134540

**Published:** 2023-06-23

**Authors:** Constantin Cristinel Girdu, Catalin Gheorghe

**Affiliations:** 1 Department of Manufacturing Engineering, Transilvania University of Brasov, Eroilor Street 29, 500036 Brasov, Romania; girdu.constantin.cristinel@unitbv.ro; 2 Department of Engineering and Industrial Management, Transilvania University of Brasov, Eroilor Street 29, 500036 Brasov, Romania

**Keywords:** entropy, hardness, laser cutting, heat affected zone, power density

## Abstract

The article presents the findings of a study on the machining of 10 mm thick Hardox 400 steel plates using the CO_2_ laser. The purpose of the investigation was to investigate the relationship between the entropy and the hardness of machined surfaces. For this purpose, a new mathematical model is established to estimate the entropy, and its influence on the hardness is determined. The mathematical model is statistically and experimentally validated. An entropy variation ΔS = −330 mJ/K between 2 K is found, causing a decrease in hardness compared to the standard value. The influences of input parameters (laser power, cutting speed, and auxiliary gas pressure) on hardness are determined. It is demonstrated that the surface hardness is strongly influenced by the auxiliary gas pressure. The combination of laser power *P* = 4200 W with gas pressure *p* = 0.45 bar at average cutting speed *v* = 1400 mm/min leads to a hardness of 38 HRC, extending the life and wear resistance of the cut parts.

## 1. Introduction

One of the modern technologies widely applied in industrial production is laser cutting. Included in the category of nonconventional technologies, laser processing is used for various technological operations, such as cutting, engraving, drilling, welding, etc. [1]. Technology has spread in recent decades due to the technical, economic, and ecological advantages it implies, the most important being quality, shape, and complexity of the generated surfaces, reduced processing time, cutting precision, small waste, lack of mechanical cutting forces, and wear of the tool [2]. These advantages and the reduced impact on the natural environment have imposed laser radiation processing as an alternative to classical processing technologies [3,4]. Currently, laser technology is applied in many fields, such as aeronautics, manufacturing industry, extractive industry, medical, and others [5,6]. Materials processed with laser are of great diversity: Ferrous, nonferrous, ceramic, plastic, and others [7,8].

The theoretical results are tested on Hardox 400 steel. The criteria that determined the choice of the material are: The wide field of use, numerous industrial applications, small number of studies carried out on this material, the physical, chemical, and mechanical properties. The main characteristics of Hardox 400 steel are: Good cold plastic deformation, corrosion resistance, high wear resistance, weldability, resistance to impact loads, high strength-to-weight ratio, fatigue resistance, and not intended for additional heat treatment. The problems reported in production are generated by the high thermal conductivity and reflectivity, aspects that influence CO_2_ or fiber laser cutting of this steel [9].

Recent research carried out on HARDOX steels has been oriented towards reducing the erosion process [10], increasing wear resistance [11,12,13], determining the correlation between the chemical constituents that lead to increased weldability [14], wear resistance in mineral abrasive environments [15], properties of welds in MAG welding [16], correlation between microstructure, and mechanical properties of welded joints [17].

To outline the current state of research, a synthesis of the articles that investigated laser cutting or Hardox steel is made. Thus, Naik and Maity analyzed the influence of several auxiliary gases (argon, air, nitrogen, and oxygen) on the machined parts of Hardox 400 [18]. Ramos et al. analyzed the state of the plate surface that influences the quality of the part when cutting with a CO_2_ laser [19]. The authors used Hardox in the analysis of steel plates of different thicknesses. For cutting Hardox steel plates, Szataniak et al. compared the influence of different cutting technologies (plasma, oxygen, laser, and waterjet) on the hardness. The authors recommended weighing steel, its thickness, and the desired cutting precision when deciding the cutting procedure [20]. 

In contrast to these directions, the work is orientated towards the thermodynamic optimization of the laser cutting process of Hardox 400 steel, a new approach for this technology. The objective of the paper is to establish a new mathematical and physical model for entropy in laser cutting and to estimate the hardness of the laser-cut surface according to the input parameters (laser power, cutting speed, and gas pressure). To explain the results, several mathematical relationships are deduced that highlight the influence of different physical parameters on the transformations that take place during laser cutting.

Metal cutting with a laser source becomes exoenergic on the alloying elements producing alpha, beta, and gamma radiation. The energy problem is approached by the method of differential and integral calculation to determine the entropy of the plate, melt, and reformed layer, aiming at the physical interpretation of the degree of ordering of the studied thermodynamic systems. The experiment brings clear results regarding the variation of hardness depending on the input parameters considered so that the cutting technology becomes more efficient.

The article is divided into sections. An overview of the fundamentals of entropy, literature, information material used, and the procedures performed is provided in the following section. The research findings and the discussions surrounding them are described in the fifth section. The research conclusions are included in the last sections.

## 2. Fundamentals of Entropy Determination

In laser cutting, the melt has a decisive role as it separates the part from the cut material. The laser delivers to the metal target an amount of elemental heat Q carried through the element dx and the area A of the heated surface during the time dt expressed by Fourier’s law [21]:

(1) $$\mathrm{dQ}=-k\cdot \frac{\partial T}{\partial x}\cdot A\cdot \mathrm{dt}.$$

Boltzmann introduced a state function called entropy S that characterizes a thermodynamic system. An elementary change in entropy due to the action of the laser on the metal target can be expressed by the relation [21]:

(2) $$\mathrm{dS}\geq \frac{\mathrm{dQ}}{T}.$$

Combining Fourier’s and Bolzman’s laws results:

(3) $$T\cdot \mathrm{dS}\geq -k\cdot \frac{\partial T}{\partial x}\mathrm{dx}\cdot \frac{A}{v}=-\frac{k\cdot A}{v}\cdot \mathrm{dT},$$

(4) $$\mathrm{dS}\geq -\frac{k\cdot A}{v}\cdot \frac{\mathrm{dT}}{T}.$$

The entropy variation is obtained by integral calculation:

(5) $$S_{2}-S_{1}=-\frac{k\cdot A}{v}\cdot \ln\frac{T}{T_{0}}$$

Taking into account the melting depth established by the Drăgănescu results [5]:

(6) $$z_{m}=2\cdot \sqrt{k\cdot \tau_{i}},$$

where S is the entropy (J/K), k is the thermal conductivity (W/mK), T is the absolute temperature (K), A the melt area on the surface equal to the laser spot area (mm^2^), τ_i_ the irradiation time (s), z_m_ the melting depth (mm), d the diameter of the laser spot (mm), T_0_ the initial temperature of the material. The entropy change is obtained:

(7) $$\Delta S=\frac{\pi \cdot z_{m}\cdot d^{2}}{8}\cdot \ln\frac{T_{0}}{T_{m}}.$$

From Equation (7), it follows that the entropy decreases when the material reaches the melting temperature, and the thermodynamic system passes from a disordered state to a more ordered one. The thermal effect of the laser spot has a circular distribution having the temperature T_m_ in the center. The molecules and atoms in the melt move in an orderly manner, a result confirmed by the decrease in entropy. The equilibrium between the melt’s weight and the centrifugal force of inertia allows the melt to keep its circular shape. Heating and melting a finite portion z_m_ will result in a cut between the part and the steel plate being blown by an assist gas jet possessing sufficient energy to destroy the melt and produce an incandescent particle explosion.

The thermophysical characteristics of the material determine the amount of energy needed to heat the metal target to a specific temperature. The elemental heat relation is expressed as follows [21]:

(8) dQ=m·c·dT,

where m is the heated metal mass (mg), c specific heat (J/kg·K), dT elementary temperature variation (K). Dividing in the Equation (8) by the temperature T, results:

(9) $$\frac{\mathrm{dQ}}{T}=m\cdot c\cdot \frac{\mathrm{dT}}{T},$$

(10) $$\mathrm{dS}=m\cdot c\cdot \frac{\mathrm{dT}}{T}.$$

Integrating from the initial temperature T_0_ to T results in an entropy change:

(11) $$S_{2}-S_{1}=m\cdot c\cdot \ln\frac{T}{T_{0}}.$$

Taking into account that T > T_0_, it follows that the entropy of the heated metal portion will increase. The result indicates that the target moves from a stable, bound, solid, ordered state to a less ordered one (constituents move disordered by an entropic increase). 

The entropy change is positive when the steel plate is heated by the laser spot. Heat-treated particles occupy several states under the Boltzmann distribution law. The chaotic motion of the molecules, under laser heating conditions, causes a local strong thermal stress. Feyman shows that matter is made up of continuously moving atoms that attract each other when they are close together [22]. Chiriac shows that low entropy occurs under conditions where the substance becomes harder [23]. When it passes into the liquid state, the metal presents an increase in entropy compared to that in the solid phase because the degree of disorder of the particles increases through heating. Plavițu shows that the change ΔS is composed of the entropy change to heat the metal (dS_1_), the entropy change due to melting the metal (dS_2_), and the entropy change to heat the incandescent metal vapor (dS_3_) [21].

(12) $$dS=\;dS_{1}+\;dS_{2}+\;dS_{3},$$

As a state function, entropy is an additive physical quantity, which is a measure that expresses the degree of disorder in a thermodynamic system. The laser power density must be adjusted so that vaporization is avoided in the cutting operations to decrease the total entropy variation. Under the action of laser radiation, it is possible to calculate the entropy difference between two different states through the metal target passes. The elementary amount of heat dQ, which heats the work area, is the entropy required to change from the solid to the liquid state. The heating process being irreversible, it follows that the entropy will increase, according to Feyman [23]. The equation of the first principle of thermodynamics becomes [21]:

(13) dQ=T·dS,

(14) T·dS=dU+p·dV,

where p is the pressure, dU represents the elementary variation of the internal energy, and dV is the volume element.

The entropy difference as a function of pressure and volume, S = S(p,V) can be written as follows:

(15) $$\mathrm{dS}\left(p,V\right)=\frac{\mathrm{dU}}{T}.$$

The variation in internal energy as a result of local laser heating is stated as follows:

(16) $${p=T\cdot \left(\frac{\partial S}{\partial V}\right)}_{T}si\;{\mathrm{dU}=T\cdot \left(\frac{\partial S}{\partial p}\right)}_{V}\mathrm{dp},$$

(17) $$\mathrm{dU}\;={T\cdot \left(\frac{\partial S}{\partial p}\right)}_{V}dp\;+{T\cdot \left(\frac{\partial S}{\partial V}\right)}_{p}\mathrm{dV}-\;p\cdot \mathrm{dV}.$$

In thermodynamics, caloric coefficients are defined as follows [21]:

(18) $${C_{p}=T\cdot \left(\frac{\partial S}{\partial T}\right)}_{p},$$

(19) $${C_{V}=T\cdot \left(\frac{\partial S}{\partial T}\right)}_{V},$$

where C_p_ and C_V_ are molar heats at constant pressure and volume:

(20) $${\left(\frac{\partial S}{\partial p}\right)}_{V}={\left(\frac{\partial S}{\partial T}\right)}_{V}\cdot \frac{\partial T}{\partial p}=T\cdot {\left(\frac{\partial S}{\partial T}\right)}_{V}\cdot \frac{1}{T}\cdot \frac{\partial T}{\partial p}=\frac{C_{V}}{T}\cdot \frac{\partial T}{\partial p}=\frac{C_{V}}{T}\cdot \frac{V}{R}=\frac{3\cdot R}{T}\cdot \frac{V}{R}=\frac{3\cdot V}{T},$$

(21) $${\left(\frac{\partial U}{\partial p}\right)}_{V}={T\cdot \left(\frac{\partial S}{\partial p}\right)}_{V}=T\cdot \frac{3\cdot V}{T}=3\cdot V,$$

(22) $${\left(\frac{\partial S}{\partial V}\right)}_{p}=\frac{C_{p}}{T}\cdot \frac{\partial T}{\partial V}=\frac{3\cdot p}{T},$$

(23) $${\left(\frac{\partial U}{\partial V}\right)}_{p}={T\cdot \left(\frac{\partial S}{\partial V}\right)}_{p}-\;p=2\cdot p.$$

Taking into account the previous relations, the elementary entropy can be established with the following relations:

(24) $$\mathrm{dS}={\left(\frac{\partial S}{\partial p}\right)}_{V}dp\;+{\left(\frac{\partial S}{\partial V}\right)}_{p}\mathrm{dV},$$

(25) $$\mathrm{dS}=\frac{3\cdot V}{T}\mathrm{dp}+\frac{3\cdot p}{T}\mathrm{dV},$$

(26) $$\frac{T}{p\cdot V}\mathrm{dS}=3\cdot \frac{\mathrm{dp}}{p}+3\cdot \frac{\mathrm{dV}}{V},$$

(27) $$d\Delta S=S_{2}-S_{1}=3\cdot R\cdot \ln\frac{p_{2}}{p_{1}}+3\cdot R\cdot \ln\frac{V_{2}}{V_{1}}.$$

Previous relations used approximations of the molar heat of liquids $C_{p}≅C_{V}$. Pressure and volume are parameters that characterize the dynamic regime of the melt. The amount of melt varies depending on how deeply the laser enters the steel plate. The internal pressure of the melt plays the role of maintaining the shape by exerting itself on the surface element of the drop. The volume of molten liquid is inconstant and practically incompressible. In the melt, the flow becomes turbulent forming eddies. The variation of entropy generally shows an increase, but there are exceptional situations in which it can decrease, and the system becomes ordered. The thermodynamic probability is expressed as follows:

(28) $$P=e^{\frac{S}{k}}=e^{\frac{3\cdot R\cdot \ln\frac{p_{2}}{p_{1}}+3\cdot R\cdot \ln\frac{V_{2}}{V_{1}}+S_{0}}{k}},$$

where S_0_ is an arbitrary constant.

A different way to determine the entropy variation is proposed starting with the intensity of the laser light at the exit of the tube and the surface of the melt. The laser has a Gaussian distribution of laser beam intensity at the tube’s exit:

(29) $$I=I_{0}{\cdot e}^{-\frac{2{\cdot r}^{2}}{a^{2}}},$$

where I_0_ is incident intensity, r is the distance from the center of the Gaussian bell to a point on the graph, a is the value of r for which the laser intensity decreases by e^2^ times compared to I_0_. This is the law that describes the variation of the intensity emitted. The situation changes when the laser interacts with the materials. Part of the incident intensity R·I_0_ is reflected, and the quantity (1 − R)·I_0_ = A·I_0_ is absorbed. In this situation, the intensity formula changes as follows:

(30) $$I=\frac{(1-R)}{4\cdot \pi }{\cdot I}_{0}\cdot e^{-\frac{2{\cdot r}^{2}}{a^{2}}},$$

where R is the reflexion coefficient of the material.

The Equation (30) explains the law of variation in the laser intensity on the melt surface. Through dI/dr derivation and then through integral calculation, the radial coordinate r in the Ox direction is obtained:

(31) $$\frac{\mathrm{dI}}{I}=-\frac{4\cdot r}{a^{2}}\mathrm{dr},$$

(32) $$r=a\cdot [\frac{\ln\frac{I_{0}}{I}}{2\cdot (1-R)}{]}^{1/2}.$$

The variation in entropy of a mass m of material that is heated to temperature T has the expression:

(33) $$\Delta S=m\cdot c\cdot \ln\frac{T}{T_{0}}=\rho \cdot \pi \cdot r^{2}\cdot \frac{z_{m}}{2}\cdot c\cdot \ln\frac{T}{T_{0}}=\frac{\rho \cdot \pi {\cdot z}_{m}\cdot a^{2}}{2\cdot (1\;-R)}\cdot \ln\frac{I_{0}}{I}\cdot \ln\frac{T}{T_{0}},$$

where ρ is probe density (kg/m^3^).

The entropy variation is dependent on the logarithm of the state parameter system or a working parameter of the laser. Based on the previous relationships, it can be stated that entropy increases, but there may be situations in which this parameter can decrease. Entropy variation is equivalent to increasing the thermodynamic probability of finding a thermal system in more states. 

The laser is a concentrated energy source that is partially converted into the elemental heat needed to heat up in a very short time and melt a very small portion of material. Estimating this small amount of energy allows entropy to be calculated. The theoretical results are consistent with the literature in the field of thermodynamics, so using physics and laser engineering concepts can improve the entropic calculation and issue ideas regarding the stability of the melting and burning reaction. From our point of view, energy consumption can be reduced along with entropy.

## 3. Literature

Synthesis of the literature begins with the identification of the works that followed the hardness of the cut surfaces during laser cutting of different steels, regardless of their class. Yurdakul et al. analyzed four responses (surface roughness, cutting time, hardness, and heat affected zone) to laser cutting of St-52 steel. The authors proposed an optimization function of the response parameter under the influence of multiple input variables grouped with the AHP method [24]. Wardhana et al. investigated the laser-cut surface quality on 316 L stainless steel (roughness, hardness, and microstructure). The findings demonstrated that variations in cutting speed had a stronger impact on surface roughness, and pressure had a more significant impact on cutting surface hardness [25]. Cutting C45 steel 6 mm thick was explored by Boujelbene et al. using a high-power CO_2_ laser. The thickness of the heat-affected zone (HAZ), the hardness of the cut surface, and the roughness were the output variables from the power and speed input factors. For the thickness and microhardness of HAZ, the authors claimed that power and speed are the main deciding factors [26].

Because the hardness is influenced by the HAZ, a synthesis of several representative papers published in recent years was performed. Miraoui et al. investigated the impact of the diameter, cutting speed, and power of the laser beam on the depth of the melted zone, the zone impacted by heat, and the microhardness of the surface [27]. Using TSM to cut 22MnB5, ultra-high-strength steel, Tahir et al. investigated the cut width and the heat-impacted zone. With a laser spot size of 0.2 mm and a wavelength of 10.6 m, a 4 kW CO_2_ laser was used to cut the samples [28]. Petković et al. analyzed the HAZ based on the laser input parameters (speed, power, and pressure) using the ANFIS method. According to the results, the speed had the greatest impact on the HAZ forecast and the gas pressure the least [29]. Rajaram et al. analyzed the effects of power and feed rate on the HAZ, surface roughness, scoring frequency, and kerf width. The experimental material was steel 4130, and the samples were obtained with a CO_2_ laser. The findings showed that power had a significant impact on cut width and HAZ size, while the impacts of speed were minor [7]. In order to assess the roughness, HAZ, and taper profile of the hole, Patel and Bhavar studied the influence of several input parameters. The steel used was EN-31, with 6 and 10 mm thicknesses. The authors established that pressure and power were critical factors for controlling HAZ and roughness [30]. Çaydaş and Hasçalık proposed an optimization model for laser cutting of St-37 steel. Cutting speed and laser power were optimized, taking into account roughness, top cut width, and HAZ width. Laser power was found to have a more pronounced effect on responses compared with cutting speed [31].

The literature survey continues with the selection of recent publications with comparisons between different types of lasers in order to assess the relevance of such research. The approach is justified because in world production, the CO_2_ laser has been gradually replaced by a fiber laser. Sotysiak et al. compared the performance of two different types of lasers [32]. The authors demonstrated that a more precise cutting surface is obtained using fiber laser on the 6 mm thickness S235JR steel. However, the purchasing decision belongs to the users, as fiber lasers have a high purchase price. Another comparative analysis that included both types of laser was done by Kubišová et al. [33]. The authors analyzed the roughness parameters R_z_ and R_a_ and found little differences between the observed parameters, although the fiber laser provides a better surface quality. A comparison of the surface quality produced by various cutting procedures (laser beam cutting, plasma cutting, and oxygen cutting) was carried out by Górka and Poloczek (2018). The material used was S960QL steel. Among the results obtained, it is noted that laser and plasma cutting determined an increase in surface layer hardness up to 490 HV [34]. Zaitsev et al. used both types of laser to cut 1.5 and 8 mm thick steel [35]. The researchers noted that both lasers have the same intensity distribution as the absorbed energy. Another conclusion of the authors was that during fiber laser cutting, the sidewalls overheat. Bursi et al. recommended laser processing to obtain parts characterized by complex geometry and high-precision processing. The authors used S355N steel as the study material, and the comparatively analyzed processes were oxygen, plasma, and laser cutting [36]. The conclusions drawn from the literature review show that CO_2_ laser research is necessary as it remains a more affordable option for many users worldwide.

For the interpretation of the entropic model calculation, we proceed to identify several papers that present aspects related to metallography. Boley et al. investigated the melt pool in the laser welding process by X-ray videography in combination with high-speed optical cameras. The metallography of the sections has been rendered by colors that provide information about length, width, and depth [37]. Gonzalez et al. treated the microstructure of a laser-welded Zry-4 sheet. The structure of the base material is granular and, by melting and cooling, the material acquires an acicular granular structure (α and β phase). A gradual change in structure is observed at HAZ due to the temperature gradient [38]. Donțu presented the image of the surface treated with the OSC10 laser. It was found that the initial structure of the sample was metallic ferrite, and by laser heating, the final structure was made up of ferrite and carbides [39]. Kusiski showed that M2 tool steel samples coated with an anti-reflective graphite layer led to an increase in HAZ and a coarser structure of the resolidified layer. Laser treatment of M2 steel in an oxygen atmosphere led to a decrease in Cr, V, and Mn content in the melted and resolidified zone [40]. Kukliski studied laser-treated samples of Monel 400 coated with boron. He found that alloying hardens the surfaces [41]. Mehrpouya showed that the microstructure of the H13 tool steel changed after laser treatment in the reworked layer, HAZ, and base metal. According to the author, the surface grains were restructured by laser irradiation [42]. Boboescu showed that during the laser welding of Dillimax500 steel plates, a keyhole portion was formed around the laser radiation, which increased in depth. After the cessation of irradiation, this portion was filled with melt. During the cooling and solidification phase, the keyhole area contained air bubbles or pores affecting the quality of the weld [43]. Drăgănescu treated 34MoCrNi15 and OLC45 steels with a CO_2_ laser. The hardened layer was found to have a martensitic structure (C-Feα) and a constant depth hardness. The 34MoCrNi15 and OLC45 cores had a bainitic-sorbitic structure (acicular-granular) and a sorbitic structure (fine lamellar structure) structure [5]. From the presented synthesis, it follows that the alloys containing atoms of C, Mo, Cr, W, Ni register changes in concentrations after laser irradiation, which means that the hardness and resistance vary. Changes in the chemical components of the alloying elements are considerable as a result of the use of different laser energies.

The literature synthesis ends with the study of representative papers related to entropy in laser processing, regardless of the processed material. Yilbas showed that heating with short laser pulses generated an unbalanced energy transfer in the metal substrate. In the electron-lattice coupling model, the volume entropy was determined based on the kinetic energy of the electrons [44]. Zanin et al. developed a permutation entropy for chaotic and complex systems [45]. The mathematical relations of entropy permutation and normalization depended on the ln and log_2_ functions of the permutation elements $\pi_{i}$. Halliday calculated the probability for N molecules to be found simultaneously in volume V, taking into account that the probability of finding a molecule depends on this volume. The author estimated the entropy S and the entropy variation ΔS of the system, taking into account the disorder parameter. In the steady state, the entropy was maximum, the most statistically probable, and in a spontaneous process, the entropy decreased [46]. Cisman showed that in the case of an isothermal process, the entropy variation was independent of the temperature and proportional to the logarithm of the volume ratio. The concept of entropy was assimilated with reduced heat, which represented the variation in the quantity of heat per degree of temperature [47]. No paper has been identified that pursues the relationship between entropy and hardness in laser cutting. Another finding is related to the lack of research to find the importance of the input parameters on the entropy or the surface hardness.

## 4. Material and Experimental Method

### 4.1. Design of Experiments

The mechanical properties of Hardox 400 steel are: Hardness H_a_ in the range (370–430) HBV, yield strength R_e_ = 1000 N/mm^2^, and tensile strength R_m_ = 1250 N/mm^2^ [48]. Hardox 400 steel is not recommended for dynamic applications. Table 1 displays the chemical composition of Hardox 400 steel.

The experiments performed on the laser cutting of Hardox 400 steel were designed according to a reduced model in which 3 planned factors interact at 3 input levels. Irradiation experiments show that parts with different geometry are produced. Through the experiences described in the paper, the surface hardness of 45 samples was determined. Four multiplications were run with the same input parameters. Then, measurements were taken to determine the hardness. The experimental data and responses were statistically processed on 45 parts to identify the combination of input parameters on hardness extreme points. Detailed analysis was carried out with the help of the microscope to establish the microstructure of the superficial layer and the dispersion of light on the particles that form the HAZ.

The 10 mm thick Hardox 400 alloy steel plate was subjected to trial tests to identify the midpoint values for each input parameter. The reduced experiment contained 3 laser cutting input parameters used by the machine software and set from the work parameters dialog box (power P = 4200 W, pressure p = 0.45 bar, and speed v = 1400 mm/min). In the cutting experiments, the focal position was f = −0.5 mm inside the material. The experimental design was created so that each independent experiment accepted combinations of hardness and influencing factors located at equidistant levels. Table 2 contains the parameters used in the CO_2_ laser cutting experiments.

**Figure 1:**
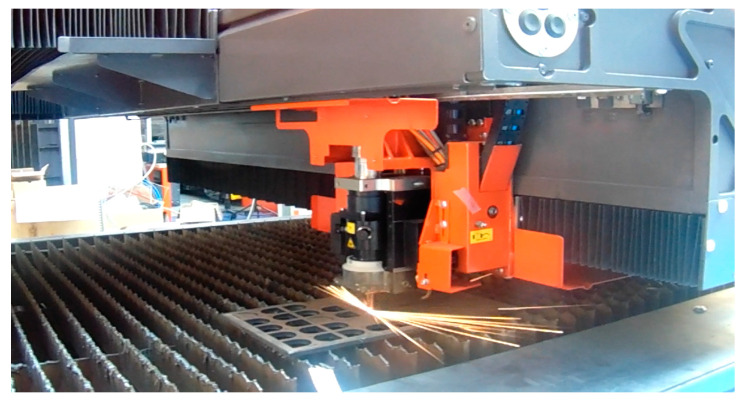
Cutting samples with Byspeed 3015 laser installation.

The piercing was carried out in pulsed mode with the following technical characteristics of the installation: Nozzle height 3 mm, power 4400 W, piercing gas pressure 0.8 bar, initial pulse frequency 100 s^−1^, the final pulse frequency 130 s^−1^, pulse width 1200 µs, focal position in piercing 4 mm. The research team interacted with the laser machine through the ByVision dialog box. The cutting head included the following technical features: Laser light-focusing lens focal length 7.5 inch, nozzle diameter 1.5 mm, nozzle type NK1515, assist gas type O_2_, standby gas pressure 1.2 bar. Figure 1 shows the laser cutting installation Laser Byspeed 3015 with a maximum power of 4400 W. 

**Figure 2:**
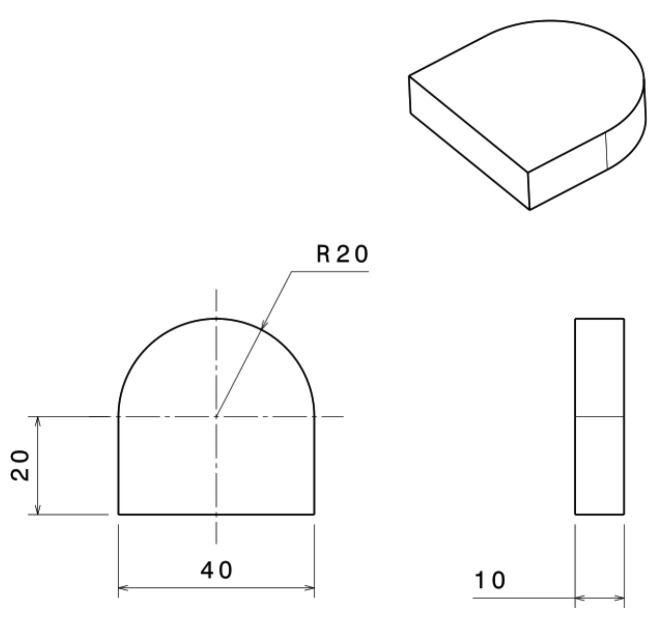
Dimensions of the workpiece (mm).

The workpiece has a geometric outline consisting of three rectilinear sides, a straight profile, and a semicircular profile, as in Figure 2. The workpiece measured 40 mm in length, 40 mm in height, and 20 mm for the radius of the semicircular profile.

### 4.2. HAZ Measurement and Microstructure

The hardness was measured on the circular profile of each part. The measuring instrument used was SonoDur 3 with a probe based on the UCI (ultrasound-contact-impedance) method. Measurements were made at 5 points/part, resulting in an average equal to the value of one H_a_ measurement. Hardness measurements were performed on the cutting area and in the base material.

**Figure 3:**
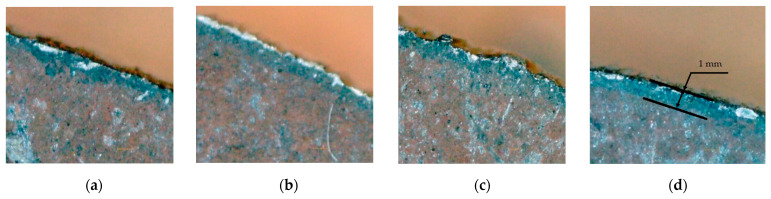
Heated affected zone. (**a**) Part 5; (**b**) Part 18; (**c**) Part 30; (**d**) Part 44.

In laser cutting, the surface layer was made by heating, partial melting, and sudden cooling. Under the micrometer thick surface layer, the HAZ zone was formed following the thermal conduction of the heat flow to the inner surface of the material [31]. Analyzing the part, a dark-colored HAZ layer ≥1 mm thick can be observed around the contour. To extend the research, a DNT portable microscope was used. The magnification of the microscope was ×250, and the memory card recorded the HAZ pictures in at least 4 parts. The parts were randomly selected to study the microstructure and HAZ layer under a microscope (Figure 3a–d).

The thickness of the white spots highlights the hardness. Part 30 has a thick blue coating, indicating that it is less affected by heat flow. The blue HAZ layer is thinner on parts 18 and 44, a result indicating an increase in hardness. Part 30 has an irregular contour due to the instability of the cutting edge. The cut edge has a lower cementite content (bright white spots). Below the HAZ, the structure consists of pearlite and ferrite. Part 44 has cementite portions over large areas in the surface layer, which increases its hardness. On the cut surface, a very thin layer of brown oxides can be observed. The HAZ layer is purple, thin at 44 and thick at 30, which shows that the resistance of the part has opposed heat penetration into the material. The non-uniformity of layer 30 is explained by the random distribution of structural components, leading to a decrease in hardness. Through the loss of cementite in the HAZ layer, the hardness decreases compared to the surface layer. Changes in part structure and physical properties are caused by the high local heating and chemical reactions of laser radiation [37]. The surface of the Hardox 400 part has chemical changes due to the oxidation reaction. In part 30, the transformations produced by the laser with a minimum of energy highlight the decrease in the hardness of the superficial layer by reducing the temperature gradient in the part. It was observed that the depth of thermal hardening was greater in part 30, which led to a decrease in the hardness. Heat flux has the main effect on the hardness drop in the HAZ compared to the laser-cut surface.

The interaction time is another laser parameter that changes the metallographic structure of the affected area. Thermal treatment is influenced by the nonuniform distribution of laser energy according to Gauss’s law. The grain density is high in the part 30 aspect, indicating the increase in strength under the HAZ. The shallow width on part 44 demonstrates high hardness. The microstructure of the surface layer in part 44 shows fine grains, which give a high microhardness compared to the other analyzed parts. In part 30, the small width indicates the decrease in hardness on the laser-cut surface. Experimental evidence shows that while the laser works, the hardness changes on the cut surface and increases in depth as it penetrates deeper into the core.

## 5. Results and Discussion

### 5.1. Results

A number of 45 parts are debited and subsequently subjected to hardness analysis. Table 3 shows the results of the experimental data (series 1) for hardness and the input variables.

Table 3 shows that the hardness points measured for the 45 samples have values in the range (32.1, 38.3) HRC. The minimum hardness value (part 21) is attained at minimum power while the pressure and speed are selected at maximum. The hardness becomes maximum (part 14) when selecting the power at the medium level, while the gas pressure is medium and the cutting speed is maximum. Comparing the running mode of the cutting parameters shows that between the minimum and maximum hardness, the cutting speed remained constant at the maximum level.

Thus, it is observed that the speed has no significant influence on hardness. The shortest interaction period between the laser spot and the steel, which has the effect of reducing local heat, may be explained physically. The pressure set antagonistically to the cutting speed has the action of reducing the heat accumulation in the slot, with the effect above hardness. The laser power selected at the average value maximizes the hardness by providing the parts with an average amount of laser energy that thermally heats the cutting area.

Following this cutting experiment, it was found that the hardness decreases on the circular side of the parts. The laser intensity and the spread of the heat front reduce the hardness of the material after cutting the three line profiles of the workpiece. Laser cutting has the effect of decreasing the hardness by dislocating the constituents due to vibrations. The surface layer is strongly influenced by the heat wave in the material and the heat flow produced by laser melting. Interference of the two thermal waves propagated by Laplace conduction and the Marangoni effect causes strong heating in the depth of the superficial layer [49]. For Hardox steel, the hardness of the parts was found to decrease, even if the cutting is better by preheating. The experimental data on the 45 samples are sufficient to find a functional relationship between the hardness and the input parameters.

The initial experiment has the lowest average hardness, the reason being that the plate temperature is equal to the ambient temperature. When cutting the replicas, the hardness is observed to increase so that replica 1 has the highest hardness, one of the reasons being preheating. A function that can analyze hardness is entropy since its variation mode can induce hardness on the cut surface. Another factor that influences hardness is the chemical composition of the surface layer. The structure of the target surface can influence the hardness variation depending on the behavior of the material upon laser absorption. The decrease in hardness by 6.2 HRC is a confidence limit so that the obtained experimental data can be processed and interpreted statistically. The average hardness of the plate is 40 HRC, so it can be seen that the laser weakens the hardness of the Hardox 400 cut surfaces. The problem is due to the time-varying electric field of the laser light. This produces another variable electric field in the metal, which, at the same time, generates a magnetic field resulting in dynamic variable induced currents that produce local heating. The melt is produced, the size and characteristics (viscosity) of which influence the hardness of the resolidified layer [50].

The range of experimentally measured hardness variations is equal to 2.67 HRC, which is 7.64% of the mean hardness equal to 34.94. The statistical error of the experiment is 2%. Since the error value is below 5%, it indicates that the hardness and error configuration points follow the Gaussian distribution. The results obtained in Table 4 demonstrate that the material, cutting parameters, and focus position are correctly chosen.

### 5.2. Hardness Analysis

The laser processing of the Hardox 400 steel led to heat treatments of the cut surfaces that have the effect of changing the physical and chemical properties. After cutting, the part acquired other characteristics after hardening the surface layer. The material is penetrated by a high value of laser light power density, which has effects on the side surface of the part in the martensitic phase. The HAZ has dimensions larger than the diameter of the laser spot. Thermal quenching is produced by sudden heating until the melting of the surface layer and cooling in a very short time to the ambient temperature, characterized by a temperature gradient. The structure is studied microscopically, and the hardness is measured. It is found that it remains constant over the quenching depth and decreases in function of the base material. Below the HAZ, hardness starts to increase with depth. These observations led the authors to carry out a statistical study. 

**Figure 4:**
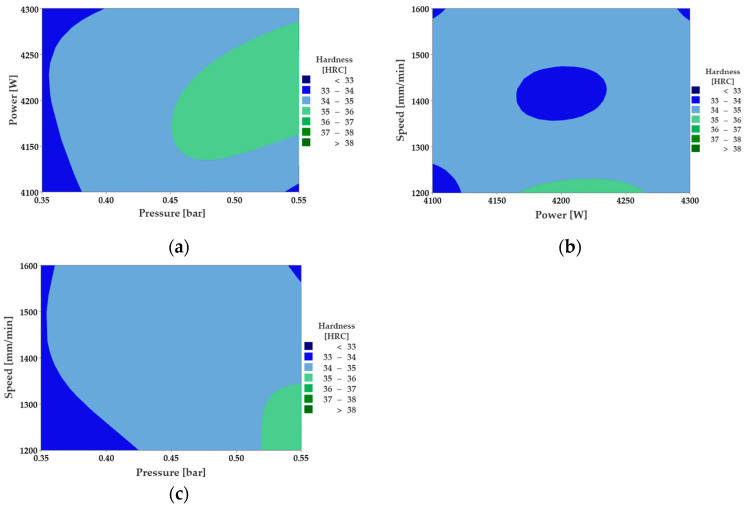
Hardness contour plot: (**a**) (*p*,*P*); (**b**) (*P*,*v*); (**c**) (*p*,*v*).

Next, the influence of the initial factors (power, pressure, and cutting speed) in the prediction of the hardness variation is followed. Contour plots from Figure 4 show the influence of two predictors simultaneously on hardness.

Contour plots estimate hardness as a function of the interaction between two parameters, while the third is constant. The surface area and color indicate the intensity of the hardness according to the values of the control parameters. Figure 4a shows that the hardness is maximum when the assist gas pressure is between medium and maximum, while the laser power has values close to the medium level. In the interaction (*p*, *P*), the pressure of the assistant gas strongly influences the hardness due to the contour surfaces that delimit the larger areas with hardness compared to Cases 4b and 4c. Figure 4b recommends that the minimum level of hardness is obtained in the center of the contour surface at medium values of laser power and speed. Figure 4c indicates that the maximum set hardness at maximum pressure can be achieved while the cutting speed becomes minimum. The contour surfaces obtained are elliptical, circular, flattened disks of various colors that predict the hardness output variable quite well. The hardness values are compared with the statistical mathematical model to check the compatibility and selection of cutting parameters.

### 5.3. Hardness Analysis

**Figure 5:**
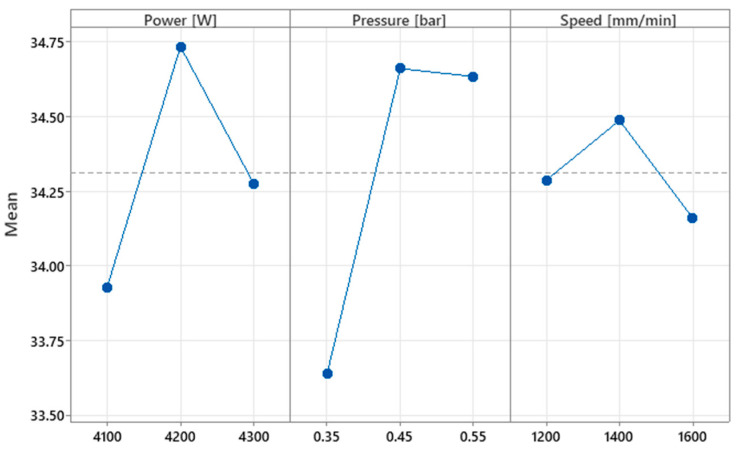
Effect of input parameters on hardness.

The experimental data are statistically analyzed to assess the effect of the cutting factors on hardness. Figure 5 shows how each parameter influences the mean of the hardness. The experiments are implemented so that the input parameters mitigate the effects of some noise factors (condition of the surface of the material, humidity of the environment, and temperature of the plate) during laser processing. The levels of each factor influence the hardness characteristic differently. Setting the power in the (4100–4200) W range has the effect of a sharp increase in hardness up to the maximum value of 34.75 HRC, where the constituents are close to each other, increasing the attraction forces. Increasing the power up to 4300 W results in a less drastic reduction in hardness.

The increased energy of the laser in excess leads to an accumulation of thermal energy, which has effects on the crowding of the constituents and repulsion between them, influencing the decrease of hardness. The pressure gas varied in the range of (0.35–0.55) bar. For the first half of the interval, a steep increase in hardness is observed. When the pressure is in the second half of the range, the hardness starts to decrease slowly. The thermochemical reaction is slower in the (0.45–0.55) bar range, which has impact of fixing constituents at very small interatomic distances, where attractive forces can increase hardness. Maximum hardness is obtained at the average level pressure of 0.45 bar. The speed between (1200–1600) mm/min had the effect of hardness dynamics in a narrow range of values (34.15–34.5) HRC. In the first half of the range, speed causes a small increase in hardness, while in the second half, it causes a slightly more pronounced decrease in hardness. At average speed, the level reached by the average response is 34.50. A gradual change in speed value from 1400 mm/min results in a decrease in hardness. It is noticed that the central values of each cutting parameter raise the average hardness. The slope between the response mean, and the control factor is the highest for the pressure, indicating that the effect size is the highest.

### 5.4. Mathematical Model for Hardness

**Figure 6:**
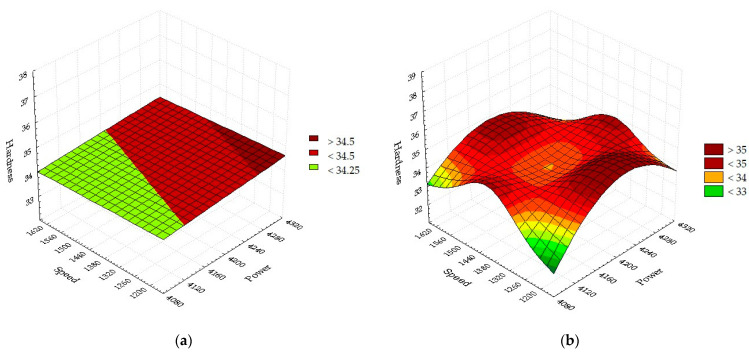
Dependence of hardness as a function of power and speed: (**a**) Linear; (**b**) Quadratic.

The response surface method (RSM) is used to produce the predictive plot. All possible combinations of laser strength and cutting speed are shown on a 2D surface (Figure 6). Under these conditions, the graph and dependence relationship between the hardness, depending on power and speed, was developed (Figure 6a). It is found that, at a maximum transfer of laser energy, the material absorbs electromagnetic radiation very well. A restricted local area is formed, which heats up strongly, melting the metal due to the transformation of the laser radiation energy into heat. In this case, the temperature created by the laser is maximum, which has an effect on the surface hardness. The interaction time between the laser and the metal target is longer at the slowest speed, resulting in a larger amount of melted material. The cut surfaces have different temperatures than the formed melt. This aspect proves that a smaller amount of heat is induced in the cut surface through the phenomenon of convection and thermal conduction compared to the liquid portion. The heat created and transferred reconfigures the atoms in the surface layer, forming a metallic network with a structure of lower hardness than initially. From a physical point of view, the atoms are further apart, and the interaction forces between the chemical constituents are reduced. Progressively changing the values of decreasing the power from 4200 W and increasing the speed from 1200 mm/min, it is observed that the hardness will decrease linearly on the flat surface. The minimum hardness of 34 HRC is obtained under maximum speed and minimum power machining conditions. The linear regression polynomial describes the effect of the factors, power (L) and speed (L). The statistically determined mathematical relationship becomes:

(34) $$H_{a}=27.4744+0.0017\;\cdot \;P-\;0.0003\cdot v.$$

Substituting *P* = 4200 W and *v* = 1200 mm/min results in H_a_ = 34.25 HRC. In the Equation (34), the laser power will increase the hardness, and the cutting speed will have the opposite effect.

The quadratic pattern H_a_(*P*,*v*) is due to the interaction between power (Q) and speed (Q). Figure 6b is similar to a hyperbolic paraboloid. Maximum hardness points are obtained under conditions at medium power with maximum or minimum speed, respectively, at maximum or minimum power with medium speed. The hardness decreases when the power is minimum, while the speed is minimum or maximum. The two graphs show a better fit between the linear and quadratic models at the minimum power, while the speed is maximum. Instead, there are differences between the linear and the quadratic model for obtaining high hardness values. Considering that the quadratic model contains more interactions of influencing factors, in the production activity, this model will be chosen because it is more accurate.

(35) $$H_{a}=-1175.5311+0.5512\cdot P+0.0706\cdot v-6.3333\cdot 10^{-5}\cdot P\cdot P-1.25\cdot 10^{-5}\cdot P\cdot v-6.5833\cdot 10^{-6}\cdot v\cdot v.$$

**Figure 7:**
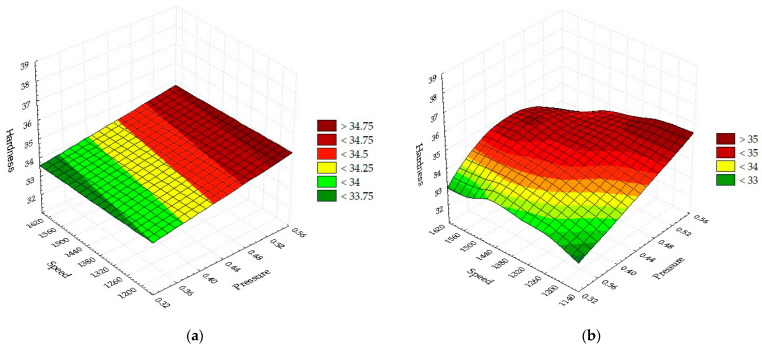
Dependence of hardness as a function of pressure and speed: (**a**) Linear; (**b**) Quadratic.

Figure 7a shows the linear model H_a_(*p*,*v*) due to the interaction between pressure (L) and speed (L). The graph is a flat surface inclined towards the minimum values of speed and pressure. Minimum hardness points are obtained for minimum speed and pressure. Maximum values are obtained when the two parameters have antagonistic values (*p* = maximum, *v* = minimum). The point of minimum hardness corresponds to a speed of 1600 mm/min and a pressure of 0.35 bar. Gradual drop in speed and increase in pressure result in hardness growth of the surface. The average global hardness calculated with the linear relationship shows a large coefficient of gas pressure, which means that in the linear interaction, pressure is the main factor. This result reinforces the conclusion that in the linear interaction between pressure and speed, the relationship between hardness and pressure is strong. The linear model shows a change in the flat surface with intensities that reflect the hardness at a fixed speed and the rolling pressure from minimum to maximum. The quadratic pressure (Q) and speed (Q) model represented in Figure 7b presents certain differences compared to the linear version. The surface formed is a hyperbolic paraboloid because of quadratic and linear interactions. According to this model, maximum hardness is obtained at minimum speeds combined with maximum pressure values. Since the quadric model is more accurate, it will be preferred in the production activity.

The linear equation describing the surface in Figure 7a has the following form:

(36) $$H_{a}=32.5194+4.9667\cdot p-\;0.0003\cdot v.$$

The quadratic equation for H_a_ in Figure 7b as a function of speed and pressure has the following form:

H_a_ = −6.8369 + 88.4667·*p* + 0.0298·*v* − 52.3333·*p*·*p* − 0.026·*p*·*v* − 6.5833·10^−6^·*v*·*v*. (37)

**Figure 8:**
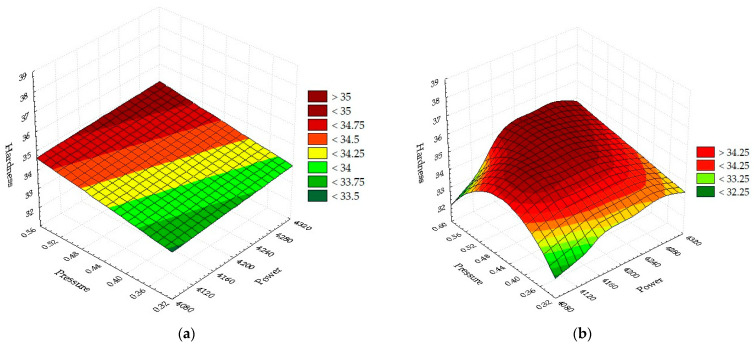
Dependence of hardness as a function of power and pressure: (**a**) Linear; (**b**) Quadratic.

Figure 8a contains the linear model H_a_(*P*,*p*). The flat surface obtained is inclined towards minimum values of power and pressure. The equation describing the surface hardness in Figure 8a is:

H_a_ = 24.7961 + 0.0017·*P* + 4.9667·*p*. (38)

In Figure 8b, the quadratic model Ha (*P*,*p*) is represented. The surface has the shape of a hyperbolic paraboloid, which is more accurate in estimating the hardness compared to the linear version. High hardness values are obtained for medium-high power and medium-high assist gas pressure values. The equation for surface 8b has the form:

H_a_ = −1076.7153 + 0.5277·*P* − 4.6333·*p* − 6.3333·10^−5^·*P*·*P* + 0.0135·*P*·*p* − 52.3333·*p*·*p*. (39)

The energy of the laser spot is focused on a very small portion of material with a diameter on the order of tens of millimeters. The energy focused in a very small volume determines how the laser interacts with the steel plate. The heat and intense light given off by the laser spot produce the cut [51]. Laser power between 4100 and 4300 W is sufficient for phase transitions of Hardox 400, causing melting and partial vaporization. The RSM method investigated the hardness when the power acts simultaneously with the pressure, while the speed is kept constant at the average level. The linear power-pressure interaction is a plane. It is formed from the set of points H_a_ due to the linear interaction P with p. For critical values antagonistic values of the hardness can be obtained at the same level of the input parameters. In the L and Q regression equations, the higher degree of the polynomial H_a_ as a function of two independent variables provides more accurate hardness. Furthermore, the value of the regression coefficient (52.3333) indicates that the cutting pressure of the gas has the strongest influence. Phenomenologically, the energy gain produces an increase in the temperature until melting, resulting in another phase transformation that will form the compact structure. Combining the laser energy with the working gas pressure can vary the amount of heat in the slot. It follows that this heat encloses the walls reforming the structure of the atoms, which renders other physical properties of the parts. The energy fusion due to the work parameters produces hardness. Depending on the configuration of the atoms on the superficial surface and the interatomic distance, surface hardening will be achieved. The attractive forces between alloy atoms (Fe, C, Si, Mn, Cr, Mo) are stronger at 4200 W power and 0.55 bar pressure. The quadratic relationship indicates that pressure plays a role in decreasing hardness. At low values of the power, it was found that the hardness varies according to a parabola with the pressure. By gradually changing the laser power, an uneven surface is found due to the linear interaction between power and pressure. The minimum power and pressure values provide a low hardness that can be used in the post-laser manufacturing process. The quadratic and linear models fit each other well, so these relationships are sufficient to obtain parts with the imposed hardness.

Next, the relationship between material hardness and entropy was evaluated. The laser radiation energy required to melt and process the part is expressed by the relation:

(40) $$E=\frac{P}{v}\cdot d,$$

where d laser spot diameter (mm). To estimate the hardness H_a_, the linear Equation (40) between power and speed is used, keeping the gas pressure constant at an average level. Substituting the power as a function of energy, the spot diameter, and the speed selected at the maximum value results in a functional relationship between energy and hardness.

(41) $$E\left(\mathrm{KJ}\right)=\frac{H_{a}-H_{\mathrm{model}}}{8.5}.$$

Taking into account the law of energy conservation E = Q, for a temperature of 373.15 K (100 degrees Celsius), H = 38 HRC results in the consumed energy E = 1240 J, and the entropy of this state S = 45.39 mJ/K. In this case, the entropy is dependent and approximated according to the laser parameters. Since power and speed define the hardness relationship at the same time, it allows us to establish a functional relationship between entropy and H_a_:

(42) $$S=\frac{Q}{T}=\frac{E\left(\mathrm{KJ}\right)}{T}=\frac{H_{a}-H_{\mathrm{model}}}{8.5\cdot T},$$

where H_model_ is the statistical model constant equal to 27.47 HRC. It follows that the entropy depends on the hardness.

The entropy of the piece increases with increasing laser energy. Substituting in Equation (42) results in the entropy values from Table 5.

## 6. Results Interpretation

### 6.1. ANOVA Analysis

The input parameters that affected the hardness were determined using the ANOVA approach. To determine how the input parameters affect the surface hardness, the coefficient R-sq was determined. Table 6 displays the ANOVA results for hardness. 

**Figure 9:**
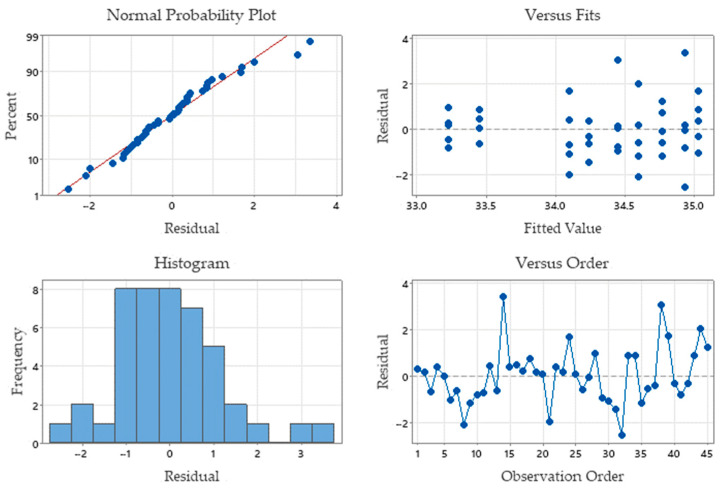
Residual plots for hardness.

A 95% level of confidence was used when using the ANOVA approach. DF represents the degrees of freedom, SS is the sum of the squares of the regression and the errors, MS is the mean of the sum of the squares, and F is the average Fischer [52]. The *p*-value should be less than or equal to the 0.05 significance level to show that one of the input parameters has a significant effect on the response [52,53]. The errors are obtained from the measured hardness relative to the value calculated as a global average. The square deviation of the error is 63.6791. Probability p = 0.050 indicates that the effect of the gas pressure on the hardness is strong. A value of F = 3.03 and p = 0.05 shows that there is a significant relationship between hardness and pressure. Therefore, the gas pressure is the main parameter. Figure 9 shows the residual values for hardness. Considered together, the residual plot tests show good agreement with hardness, which satisfies the ANOVA results.

As a result of the high standard deviation, S = 1.29451, the Gaussian distribution graph appears to be flattening. The absolute difference between the measured surface hardness value and the one determined by mathematical regression is taken into account when calculating the residual error. The 45 experimental points’ cloud is quite close to the regression line, indicating the accuracy of the statistical findings. The components are made by melting, heating, and quickly cooling. As a result of these events, the standard deviation rises, and the errors are distributed at a level of 5%. Laser energy and heat can cause errors. In the ranges between (−2, 0) and (0, 0), residual errors are the most prevalent. Information regarding the distribution of errors is available in the residual error histogram. The majority of data near the mean on the right and left do not follow the Gaussian curve, respectively. The hardness error plots for each part are shown as dots. They are randomly distributed or grouped to interpret error cases. The part error graph shows outliers on parts 8, 14, 21, 32, and 38. Cutting regimes for these parts where laser cutting will be avoided are being studied. 

**Figure 10:**
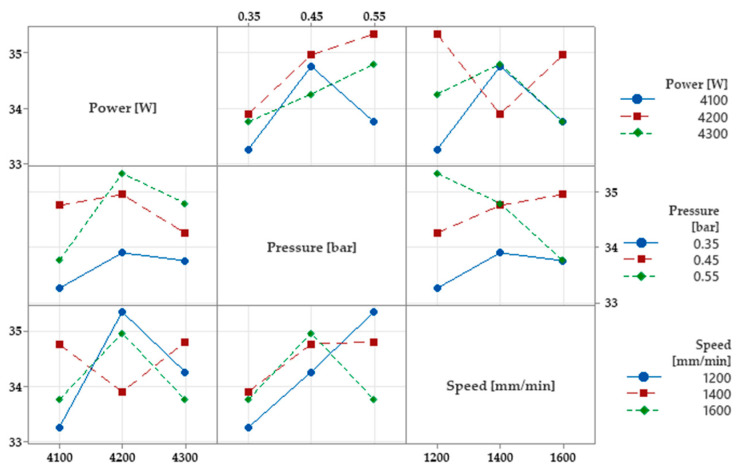
Interaction plot for Ha (HRC).

Figure 10 shows the interaction of each input parameter with the hardness. Based on the diagrams, the optimal combinations of parameters can be chosen for each average hardness value. If the pressure *p* = 0.55 bar, *P* = 4300 W the average hardness of the material increases. When *v* = 1200 mm/min with laser power *P* = 4200 W is selected, the hardness increases. The combined effects of power *P* = 4200 W with pressure *p* = 0.55 bar, which will give hardness over 35 HRC, respectively, average power with minimum speed. These interaction diagrams are important because they indicate select input factors that combine at the same time to produce a surface of a certain hardness.

### 6.2. Newton Interpolation Method to Check the Significant Factor (O_2_ Pressure)

The Newton interpolation polynomial, which demonstrates a functional relationship between the hardness and pressure of the assistant gas, is used to validate the experimental results (Table 7).

The Newton function approximating the hardness of the surface is rendered by the interpolation polynomial:

(43) $$H_{a}=f\left(x_{1}\right)+f\left(x_{1},x_{2}\right)\cdot \left(x-x_{1}\right)+f\left(x_{1},x_{2},x_{3}\right)\cdot \left(x\;-x_{1}\right)\cdot \left(x\;-x_{2}\right).$$

The hardness approximation polynomial is obtained as a function of the pressure:

(44) $$H_{a}=-110\cdot p^{2}+88\cdot p-16.5.$$

The high values of the interpolation polynomial coefficients indicate a strong relationship between hardness and gas pressure. This result validates the statistical results showing that pressure is the main influencing factor in the formation of the hardness of the surface. Adjusting the pressure of the cutting gas to the value *p* = 0.45 bar, it is obtained a part with a hardness of 34.5 HRC. This value is close to the one obtained with the global interpolation defined with the Newton interpolation polynomial, H_a_ = 33.4 HRC. The error obtained between the calculated and measured values is below 1%, therefore, the input data approximate the hardness well enough.

### 6.3. Entropy Variation Verification

Hardox 400 steel is a paramagnetic metal, where the Curie constant is C = 1.14 × 10^−4^ m^3^ × K/kg. The material obeys the Curie–Weiss equation, where the connection is made between the total magnetic moment M and the magnetic field strength H [54]:

(45) $$M=C\cdot \frac{H}{T},$$

where H (A/m) is the magnetic intensity, and T is the temperature (K). Taking these data into account, we attempt to model the entropy S = S(T,H). For this purpose, the differential is calculated:

(46) $$\mathrm{dS}=\left(\frac{\partial S}{\partial T}\right)\mathrm{dT}+\left(\frac{\partial S}{\partial H}\right)\mathrm{dH}.$$

The magnetic moment can be described in differential forms, M = M(T,H) by the relation:

(47) $$\mathrm{dM}=\left(\frac{\partial M}{\partial T}\right)\mathrm{dT}+\left(\frac{\partial M}{\partial H}\right)\mathrm{dH},$$

The specific heat in a constant magnetic field can be calculated as follows:

(48) $$C_{H}=T\cdot \frac{\partial S}{\partial T},$$

where C_H_ is specific heat.

Taking into account the Curie relation and the partial derivative:

(49) $$\left(\frac{\partial M}{\partial T}\right)=-C\cdot \frac{H}{T^{2}},$$

(50) $$\left(\frac{\partial M}{\partial H}\right)=\frac{C}{T}.$$

Define the term:

(51) $$\left(\frac{\partial S}{\partial H}\right)=\mu_{0}\cdot \left(\frac{\partial M}{\partial T}\right)=-\mu_{0}\cdot C\cdot \frac{H}{T^{2}},$$

where $\mu_{0}=4\pi \times 10^{-7}\;H/m.$

Under these conditions, the entropy can be estimated from the intensity of the magnetic field and the specific heat:

(52) $$\mathrm{dS}=\frac{C_{H}}{T}\mathrm{dT}\;-\mu_{0}\cdot C\cdot \frac{H}{T^{2}}\mathrm{dH}.$$

In the case of an isothermal magnetization process (dT = 0), the entropy is:

(53) $$\mathrm{dS}={-\mu }_{0}\cdot C\cdot \frac{H}{T^{2}}\mathrm{dH}.$$

By integration, it is obtained:

(54) $$\int_{S_{1}}^{S_{2}}\mathrm{dS}=\int_{H_{1}}^{H_{2}}{-\mu }_{0}\cdot C\cdot \frac{H}{T^{2}}\mathrm{dH}.$$

The entropy change will be as follows:

(55) $$\Delta S={-\mu }_{0}\cdot C\cdot \frac{H_{2}^{2}-H_{1}^{2}}{{2\cdot T}^{2}}=-\frac{\mu_{0}}{2\cdot C}\cdot \left(M_{2}^{2}-M_{1}^{2}\right).$$

The Hardox 400 magnetization can be interpreted using the entropy variation, taking into account μ_0_ the vacuum magnetic permeability, C = 1.14 × 10^−4^ m^3^·K/kg, from temperature T_2_ = 3K to T_1_ = 1 K, H = 0.8 × 10^6^ A·m. The final magnetic moment M_2_ = 91.2 A·m^2^, and the initial magnetic moment M_1_ = 30.4 A·m^2^. It follows that the change in entropy is negative ∆S < 0, the steel mass resolidified by stopping the laser action gives off heat during the magnetization and hardening process [54].

The magnetic field has effects on the hardness decrease. During the magnetization process, Hardox steel loses its hardness characteristic. Hardox 400 paramagnetism increases the degree of magnetization when it is attacked by the laser. Table 8 shows the values resulting from the magnetic and entropic survey.

## 7. Conclusions

The physical context in this research is the HAZ and the entropy variation to describe how the hardness of the obtained surfaces can vary. The theoretical and experimental research in this study lead to the following conclusions:

- The paper contains a new calculation method for entropy, starting from the estimation of the elementary variation of the heat in the melt, the heat transfer from the melt to the walls, and the elementary heat given up by the part in the resolidification process;
- Linear (L) and quadratic (Q) models are used to observe hardness. A plane or quadric surface serves as the analysis’ response surface for hardness;
- Entropy increases when heat transfer occurs from the melt to the surface layer of the part, increasing the degree of thermal agitation of the atomic particles;
- Entropy decreases when the part resolidifies, which shows that the part orders its constituents. In this situation, the hardness of the superficial surface decreases. The entropy variation of the part is −330 mJ/K between 2 K, where the part has given up about −100 J of heat at a temperature of 300.15 K (27 degrees Celsius);
- The minimized laser energy decreases the hardness of the parts by the heat distributed in the local area, which has the effect of increasing the interatomic distances resulting in the decrease of the forces between the atoms;
- The propagation of the melting front due to the heat flow is different for each part;
- HAZ is influenced by the main parameters of the cut, the unsteady thermal flow of the cut, and the physical properties of the material. High surface hardness is obtained under the conditions *P* = 4200 W, *p* = 0.45 bar, and *v* = 1400 mm/min.;
- The pressure-power graph recommends cutting parameter values *p* = 0.35 bar and *P* = 4100 W for H_a_ < 33 HRC;
- The regression graph between pressure and power shows a low hardness <32.25 HRC in the combination of *p* = 0.35 bar and *P* = 4100 W, which means that selecting the parameters at the minimum level generates the lowest hardness;
- ANOVA analysis shows that the most influential parameter on the hardness of Hardox 400 parts is gas pressure, followed by power and speed;
- The Newton interpolation method of hardness versus assist gas pressure confirms the ANOVA statistic that hardness is strongly related to cutting gas pressure;
- For the manufacture of parts with a hardness of 300 HB, the combination of laser power *P* = 4100 W, assistant gas pressure *p* = 0.35 bar, and cutting speed *v* = 1400 mm/min is recommended.

## Figures and Tables

**Table 1:** Chemical composition.

Alloying Element	C	Si	Mn	P	S	Cr	Ni	B	Mo
%	0.14	0.69	1.60	0.024	0.01	0.3	0.25	0.004	0.25

Source: [48].

**Table 2:** Working parameters.

Parameter	Level		
Laser power (W)	4100	4200	4300
Gas pressure (bar)	0.35	0.45	0.55
Cutting speed (mm/min)	1200	1400	1600

Source: Author’s own calculations.

**Table 3:** Hardness results.

Experiment	Power [W]	Pressure [bar]	Speed [mm/min]	Hardness [HRC]
1	4100	0.35	1200	33.5
3	4100	0.55	1600	33.4
4	4200	0.35	1400	34.6
5	4200	0.45	1600	34.9
6	4200	0.55	1200	34
7	4300	0.35	1600	32.8
8	4300	0.45	1200	32.5
9	4300	0.55	1400	33.6
10	4100	0.35	1200	32.4
11	4100	0.45	1400	33.7
12	4100	0.55	1600	34.5
13	4200	0.35	1400	33.6
14	4200	0.45	1600	38.3
15	4200	0.55	1200	35.4
16	4300	0.35	1600	33.9
17	4300	0.45	1200	34.8
18	4300	0.55	1400	35.5
19	4100	0.35	1200	33.4
20	4100	0.45	1400	34.5
21	4100	0.55	1600	32.1
22	4200	0.35	1400	34.6
23	4200	0.45	1600	35.1
24	4200	0.55	1200	36.7
25	4300	0.35	1600	33.5
26	4300	0.45	1200	34
27	4300	0.55	1400	34.7
28	4100	0.35	1200	34.2
29	4100	0.45	1400	33.5
30	4100	0.55	1600	33
31	4200	0.35	1400	32.8
32	4200	0.45	1600	32.4
33	4200	0.55	1200	35.9
34	4300	0.35	1600	34.3
35	4300	0.45	1200	33.4
36	4300	0.55	1400	34.2
37	4100	0.35	1200	32.8
38	4100	0.45	1400	37.5
39	4100	0.55	1600	35.8
40	4200	0.35	1400	33.9
41	4200	0.45	1600	34.1
42	4200	0.55	1200	34.7
43	4300	0.35	1600	34.3
44	4300	0.45	1200	36.6
45	4300	0.55	1400	36

Source: Author’s own calculations.

**Table 4:** Measured average hardness values.

Experiment	Initial	Replica				Average
		1	2	3	4	
Hardness [HRC]	33.76	36.03	35.00	34.19	35.73	34.94

Source: Author’s own calculations.

**Table 5:** Entropy results.

Part	21	18	14
Hardness [HRC]	32.1	35.5	38.3
Entropy [mJ/K]	20.21	34.85	46.93

Source: Author’s own calculations.

**Table 6:** ANOVA results.

Source	DF	SS	MS	F	p	Remark
V	2	4.9124	2.4562	1.47	0.244	Insignificant
p	2	10.1391	5.0696	3.03	0.050	Significant
P	2	0.8138	0.4069	0.24	0.786	Insignificant
Error	38	63.6791	1.6758			
Total	44	79.5444				
R-Sq. = 19.95%, R-Sq. (Adj.) = 7.31%, S = 1.29451						

Source: Author’s own calculations.

**Table 7:** Experimental data in the Newton interpolation method.

Number	Pressure (Bar)	Hardness (HRC)
1	*p*_1_ = 0.35	y1 = f(*p*_1_) = 33.5
2	*p*_2_ = 0.45	y2 = f(*p*_2_) = 34.6
3	*p*_3_ = 0.45	y3 = f(*p*_3_) = 33.4

Source: Author’s own calculations.

**Table 8:** Values obtained.

Temperature T_1_	Temperature T_2_	Total magnetic moment M_1_	Total magnetic moment M_2_	Entropy variation S_12_	Heat given Q_12_
3 K	1 K	30.4 A∗m^2^	91.2 A∗m^2^	−330.6 mJ/K	−99.1 J

Source: Author’s own calculations.

## Data Availability

The data presented in this study are available on reasonable request from the corresponding author.
